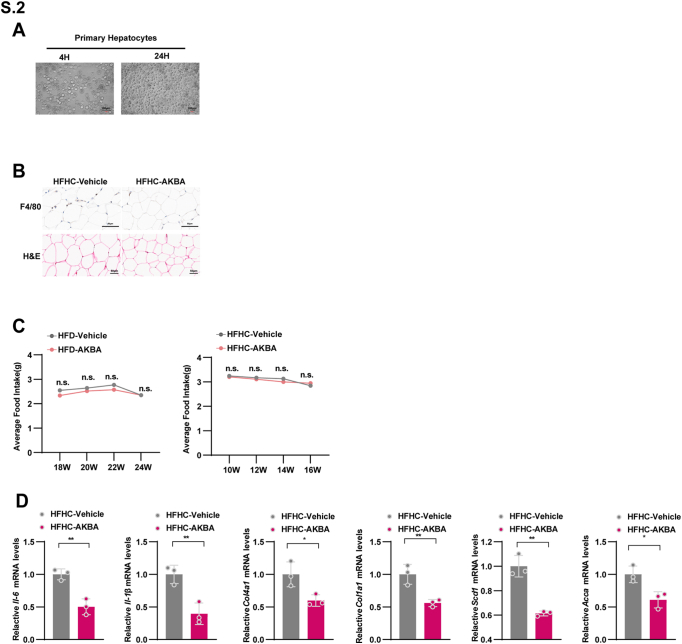# Acetyl-11-keto-β-boswellic acid alleviates hepatic metabolic dysfunction by inhibiting MGLL activity

**DOI:** 10.1016/j.jlr.2025.100812

**Published:** 2025-04-17

**Authors:** Kai Luan, Yuhong Fan, Qin Yang, Hailong Yang, Zelin Zhou, Ju Huang, Zhigang She, Toujun Zou, Hui Xiong, Zhinan Mei

**Affiliations:** 1 School of Pharmaceutical Sciences, South-Central Minzu University, Wuhan, China; 2 State Key Laboratory of New Targets Discovery and Drug Development for Major Diseases, Gannan Innovation and Translational Medicine Research Institute, Gannan Medical University, Ganzhou, China; 3 Department of Cardiology, Renmin Hospital of Wuhan University, Wuhan, China; 4 College of Plant Science and Technology of Huazhong Agriculture University, Wuhan, China

**Keywords:** nonalcoholic fatty liver disease, nonalcoholic steatohepatitis, acetyl-11-keto-beta-boswellic acid, monoacylglycerol lipases

## Abstract

Metabolic abnormalities have emerged as a central pathogenesis in various metabolic diseases, particularly nonalcoholic fatty liver disease (NAFLD) and its associated complications of obesity and insulin resistance. Despite this, effective pharmaceutical treatments for NAFLD-related metabolic disorders remain limited. In this study, we identified acetyl-11-keto-beta-boswellic acid (AKBA), a natural compound isolated from the gum resin of *Boswellia carterii*, showing robust capacity against NAFLD as well as its related body weight gain and insulin resistance. Our findings demonstrate that the beneficial effects of AKBA on metabolic disorders are largely dependent on its direct interaction with monoacylglycerol lipase (MGLL) in hepatocytes. In vivo experiments using a high-fat and high-cholesterol (HFHC) diet-induced NAFLD mouse model revealed that AKBA effectively mitigated both the progression of NAFLD and associated metabolic dysfunctions. Proteomic and RNA sequencing analyses further elucidated that AKBA attenuates key pathways related to lipid accumulation, inflammation, and fibrosis. Mechanistically, AKBA was found to directly target MGLL in hepatocytes, inhibiting its activity in hydrolyzing monoacylglycerols. Structural analyses revealed that AKBA binds specifically to the GLU60, MET64, THR279, and PHE283 residues of MGLL. Importantly, AKBA showed no additional therapeutic effect in MGLL-deficient models, underscoring the crucial role of MGLL in mediating AKBA's therapeutic action. In conclusion, our study identifies AKBA as a novel and potent MGLL inhibitor and suggests that it holds promise as a therapeutic candidate for NAFLD and related metabolic diseases. This research highlights the potential of natural compounds in the development of targeted treatments for metabolic disorders.

Metabolic homeostasis is intricately regulated by multiple tissues to maintain a stable internal environment, with disruptions in key metabolic processes—such as glucose metabolism, lipid metabolism, and insulin signaling—playing a central role in the pathogenesis of various chronic diseases (1, 2, 3). Among these, non-alcoholic fatty liver disease (NAFLD) has emerged as one of the most prevalent and concerning metabolic disorders, contributing significantly to the global health burden. The development of NAFLD is driven by multiple factors, including lipid metabolism dysregulation, insulin resistance, oxidative stress, mitochondrial dysfunction, altered adipokine secretion, and disturbances in the intestinal microbiome (4, 5, 6). Clinically, NAFLD spans a spectrum of phenotypic manifestations, from simple hepatic steatosis to non-alcoholic steatohepatitis (NASH), advancing to liver fibrosis, cirrhosis, and ultimately hepatocellular carcinoma (7).

Despite growing awareness of the need for effective therapies to treat NAFLD and its associated metabolic disturbances, progress in developing targeted treatments remains limited (8, 9). Although clinical research has surged in recent years, only a single drug, Resmetirom—a thyroid hormone receptor β-selective agonist—has been approved by the U.S. Food and Drug Administration (FDA) for NASH treatment. However, its efficacy in achieving NASH resolution or improving fibrosis remains suboptimal, with clinical trials showing less than 30% success in meeting primary end points (10). Consequently, there is an urgent need to discover novel therapeutic targets and drugs that can intervene in the complex, multi-faceted progression of NAFLD.

Traditional Chinese Medicine (TCM) has garnered increasing attention for its potential to address metabolic abnormalities, with natural compounds offering promising advantages such as multi-target actions, favorable safety profiles, antioxidant and anti-inflammatory effects, modulation of the gut microbiota, and the ability to provide personalized treatment options (11, 12). Among the numerous bioactive compounds in TCM, terpenoids are particularly noted for their potent anti-inflammatory properties. Several extracts from *Boswellia serrata* and other Boswellia species have demonstrated efficacy in promoting hepatocyte dynamics, alleviating liver injury, and correcting glucose and lipid metabolism disorders (13, 14, 15, 16, 17, 18). Acetyl-11-keto-β-boswellic acid (AKBA), a major active compound isolated from Boswellia carterii gum resin, has shown significant anti-inflammatory effects through multiple mechanisms (19, 20, 21). Despite its promising therapeutic potential, the specific molecular targets of AKBA and its role in maintaining metabolic homeostasis remain largely unexplored.

This study aims to investigate the therapeutic effects of AKBA on NAFLD and uncover the underlying molecular mechanisms involved. Through comprehensive in vitro and in vivo analyses, we demonstrate that AKBA effectively reduces hepatic lipid accumulation and inflammation via direct interaction with monoacylglycerol lipase (MGLL), suggesting that AKBA may represent a promising, side-effect-free therapeutic candidate for the treatment of NASH. These findings not only provide insights into the molecular basis of AKBA's action but also highlight its potential as a natural therapeutic agent to address the growing global burden of NAFLD and related metabolic disorders.

## Materials and Methods

### Reagents and materials

AKBA (AF20080303, purity: 98%) was purchased from Ai Fa Biotechnology Company. The Cell Counting Kit-8 (CCK8, B34304) were obtained from Selleck. Fetal bovine serum (FBS, 10,099-141) and Dulbecco′s modified Eagle′s medium (DMEM, high glucose, C11995500BT) were sourced from Gibco. Palmitic acid (PA; P0500), oleic acid (OA; O-1008-5G), BSA (BAH66-0100; Equitech Bio) and heptadecanoic acid (Hep, H3500) were purchased from Sigma-Aldrich. 2-Oleoyl glycerol (2-OG, 3443-84-3) was purchased from Avanti Polar Lipids.

### Cell viability analysis

Primary hepatocytes were seeded in 96-well plates and allowed to adhere. Following adhesion, the cells treated with increasing concentrations of AKBA (20, 40, 60, 80, 100, and 120 μM) for 14 h. After treatment, Cell Counting Kit-8 (CCK-8) reagent was added to each well according to the manufacturer's instructions. The cells were then incubated at 37°C for 2 h. Absorbance was measured at 450 nm using a microplate reader (Synergy h1, BioTek).

### Cell culture and treatment

Primary hepatocytes were cultured in DMEM supplemented with 10% FBS and 1% P/S, and incubated at 37°C and 5% CO_2_. A 200 mM oleic acid (OA) stock solution was prepared by dissolving OA in 0.01 M NaOH at 72°C for 2–3 h. Similarly, a 20 mM palmitic acid (PA) stock solution was generated by dissolving PA powder in 0.01 M NaOH at 72°C for 1–2 h. To create the final palmitic acid/oleic acid (PO) solution (OA:PA = 2:1), 1.25 ml of the 20 mM PA stock solution and 0.25 ml of the 200 mM OA stock solution were mixed with 1 ml of 25% BSA. This mixture was then added to 50 ml of DMEM and incubated at 37°C for 30 min prior to use. Primary hepatocytes were cultured in PO medium for 18 h with or without a concentration of AKBA (30 μM or 60 μM). Resmetirom (60 μM) was used as positive control.

### Cellular Oil Red O staining

After washing the cells twice with phosphate-buffered saline (PBS), they were fixed in 4% paraformaldehyde at room temperature for 20 min. Following fixation, the cells were washed again twice with PBS and then immersed in 60% isopropanol. The cells were subsequently stained with freshly prepared Oil Red O (ORO) solution for approximately 60 s. After staining, the cells were washed twice with PBS to remove excess dye. The red-stained lipid droplets were observed and imaged under a light microscope (Olympus, DP80) at 40× magnification.

### Animal experiments

The NASH and NAFLD model were established based on previous research (22). Seven-week-old male C57BL/6J mice were housed in a specific pathogen-free (SPF)-environment with controlled conditions (55%–60% relative humidity, 20 ± 3°C, and a 12-h light/dark cycle) and provided free access to water. Mice in the control group were fed a normal chow (NC) diet, and AKBA (50 mg/kg) was administered daily via intragastric gavage along with the NC diet. For the NASH model, mice were fed a high-fat, high-cholesterol (HFHC) diet (44% carbohydrates; 42% fat; 14% protein; 2% cholesterol; TP26304; TrophicDiet, Nantong) for 16 weeks. Resmetirom (10 mg/kg) and AKBA (25 mg/kg; 50 mg/kg) were administered daily via intragastric gavage during the last 8 weeks of the study. For the NAFLD model, mice were fed a high-fat diet (HFD) (60% fat, 20% carbohydrate, 20% protein; D12492, Research Diets) for 24 weeks. AKBA (25 mg/kg; 50 mg/kg) was administered daily via intragastric gavage during the final 8 weeks.

To investigate the role of MGLL in AKBA-mediated improvement in NASH, *Mgll*^*−/−*^ mice were fed the HFHC diet for 16 weeks, as previously described. All animal experiments were approved by the Animal Care and Use Committee of Renmin Hospital of Wuhan University (approval no: WDRM-20230307A) and the Animal Ethics Committee of South-Central Minzu University (approval no: 2022-scuec-055). The study was conducted in accordance with the National Research Council's Guide for the Care and Use of Laboratory Animals.

### Biochemical detection

Serum levels of alanine aminotransferase (ALT), aspartate aminotransferase (AST), total cholesterol (TC), Triglyceride (TG) and low-density lipoprotein-cholesterol (LDL-c) were measured using an automatic biochemical analyzer (7,020. Hitachi, Japan). Glucose tolerance tests (GTT) were performed at the indicated time points. Liver TG levels were determined using a diagnostic kit (Jian Cheng Institute of Biotechnology) according to the manufacturer's instructions.

### Histological analysis and immunohistochemistry

Liver tissue was embedded in optimal cutting temperature (OCT) compound and cut into 8 μm thick sections for ORO staining. Additionally, liver tissue was embedded in paraffin and sectioned into 5 μm thick slices for hematoxylin and eosin (H&E) staining or picrosirius red (PSR) staining. For immunohistochemistry, liver sections were stained with an anti-CD11b antibody (BM3925, 1:12000; Boster). The sections were first blocked with 10% bovine serum albumin (BSA) at 37°C for 1 h to prevent nonspecific binding. After blocking, the primary antibody was applied, and the sections were incubated overnight at 4°C. Following primary antibody incubation, the sections were re-warmed at 37°C for 30 min, washed three times with PBS (3 min per wash), and then incubated with secondary antibodies (PV-9001, ZSGB Biotech) at room temperature for 1 h. Immunohistochemical staining was visualized using a 3,3′-diaminobenzidine (DAB) substrate kit (ZLI-9018, ZSGB Biotech), and the sections were counterstained with hematoxylin. Images were acquired using a light microscope (ECLIPSE 80i. Nikon), and the relative stained area was quantified using Image-Pro Plus 6.0 software. H&E staining was used to evaluate the NAFLD activity score (NAS) system.

### RNA sequencing analysis

Total RNA was extracted from liver tissue of mice fed the HFHC diet and treated with AKBA. RNA sequencing was performed using the BGISEQ-500 RS platform, with 50 bp paired-end reads. The reads were aligned to the Ensembl Mouse 169 reference genome (mm10/GRCm38) using HISAT2 software (version 2.1.0). Gene expression levels were quantified by calculating raw gene counts with StringTie software (version 1.3.3 b). The raw count matrix was subsequently normalized using DESeq2 software (version 1.20.0).

### Proteomic analysis

Proteomic analysis was performed using mass spectrometry in data-dependent acquisition (DDA) mode. Peptide abundances in the HFHC diet and AKBA treatment groups were quantified using unlabeled quantitative methods. Differential abundance of peptides between the HFHC and AKBA-treated groups was analyzed for statistical significance using a *t* test. Proteins with a *P*-value <0.05 were considered differentially expressed.

### Protein expression and purification

The N-terminal 6×His fusion protein of the mouse MGLL domain was expressed using the pET28a vector. Recombinant plasmids were transformed into *Escherichia coli* BL21 (DE3) cells for protein expression. The cells were cultured to an optical density at 600 nm (OD600) of 0.6, then induced with 0.5 mM IPTG and incubated at 18°C for 16 h. After induction, the cells were harvested by centrifugation at 4°C and lysed using lysis buffer. The expressed proteins were purified using His affinity chromatography with a column pre-equilibrated with buffer (50 mM Tris, 0.15 M NaCl, 8 M urea, pH 8.0). The purified proteins were then eluted and dialyzed into PBS for further use.

### Isothermal titration calorimetry

Isothermal titration calorimetry (ITC) was performed at 25°C using a MicroCal ITC200 microcalorimeter. The proteins were dialyzed thoroughly against a buffer containing 5% DMSO and PBS. Prior to injection, the syring was filled with MGLL protein at a concentration of 24 μM, and the sample cell was filled with AKBA at a concentration of 250 μM. The heat of dilution was corrected by performing a reference titration of the ligand in the same buffer. The syringe agitation rate was set to 750 rpm. Thermodynamic data were analyzed using MicroCal Origin 7.0 software.

### Quantitative PCR

Total RNA was isolated from liver tissue using RNAiso Plus Reagent (Takara, ALE0661A). The RNA was then reverse transcribed into cDNA using the RevertAid First Strand cDNA Synthesis Kit (Takara, K1622). Real-time quantitative PCR (qPCR) was performed using SYBR Green Master Mix (Q121, Vazyme Biotech Co. Ltd.).

### Western blot

Total protein was extracted from liver tissue using RIPA buffer and quantified using a BCA protein assay kit (YC23225, Thermo Scientific). Protein concentrations were determined according to the manufacturer's instructions. The following primary antibodies were used: Anti-MGLL antibody (sc-398942, 1:200), anti-β-Actin monoclonal antibody (AC026, 1:10000, China) was purchased from Abclonal. Horseradish peroxidase (HRP)-conjugated secondary antibodies were used for detection. Antibody dilutions (P0023A) was purchased from Beyotime Biotechnology.

### Molecular docking

The crystal structure of MGLL (Protein Data Bank ID: 3HJU) was retrieved from the Protein Data Bank and subjected to energy minimization to serve as the receptor structure for molecular docking. The AKBA substrate was constructed, hydrogenated, and then optimized using the molecular orbital package. Molecular docking was performed using Autodock software, with the active site defined within the docking box and a docking time set to 100. All other parameters were set to their default values.

### Coimmunoprecipitation assay

Streptavidin magnetic beads (P2151, Beyotime) were incubated with primary hepatocyte lysates infected with an MGLL-overexpression adenovirus or 293T cell lysates transfected with the mutant plasmid for 1 h at 4°C. Following incubation, the beads were washed with IP buffer to remove unbound proteins. For antibody incubation, the beads were resuspended in IP buffer containing the appropriate primary antibody and incubated at 4°C for 2 h or overnight. After incubation, the beads were washed again with IP buffer to remove unbound antibody. The bound and total proteins were then eluted and analyzed by Western blotting.

### Enzyme assays

MGLL activity was measured using a method adapted from previous research (23). After 24 h of adenovirus infection, primary hepatocytes were collected and homogenized in 50 mM Tris-Cl (pH 8.0). The homogenate was centrifuged at 1,000 *g* for 10 min at 4°C. The supernatant was transferred to a new tube, and the protein concentration was determined using a BSA protein assay kit (YC23225, Thermo Scientific). To assess MGLL activity, 1 μg of protein was incubated with 2-OG substrate in MGLL reaction buffer in a final volume of 0.2 ml for 30 min at 37°C. The reaction was terminated by adding 0.2 ml of cold methanol heptadecanoic acid. Lipids were extracted using chloroform and water. LC/MS analysis of the samples was performed using an Agilent Eclipse Plus C18 column (2.1 × 100 mm, 1.8 μm) with a flow rate of 0.4 ml/min for 4 min. The mobile phase consisted of 100% methanol (A) and 100% water (B), each containing 5 mM ammonium acetate and 0.1% formic acid. The column temperature was maintained at 40°C. The products of interest (oleic acid, m/z = 281 and heptadecanoic acid, m/z = 269) were monitored as standards, and peak areas were quantified using LC-MS software.

### Statistical analysis

Data are shown as means ± standard deviation (SD). Statistical significance was determined using one-way analysis of variance (ANOVA) followed by Tukey's multiple comparison test, or by Student's *t* test, as appropriate. All analyses were performed using GraphPad Prism 8 (GraphPad). A *P*-value < 0.05 was considered statistically significant.

## Results

### AKBA reduces PO-induced metabolic disorders in primary hepatocytes

**Fig. 1:**
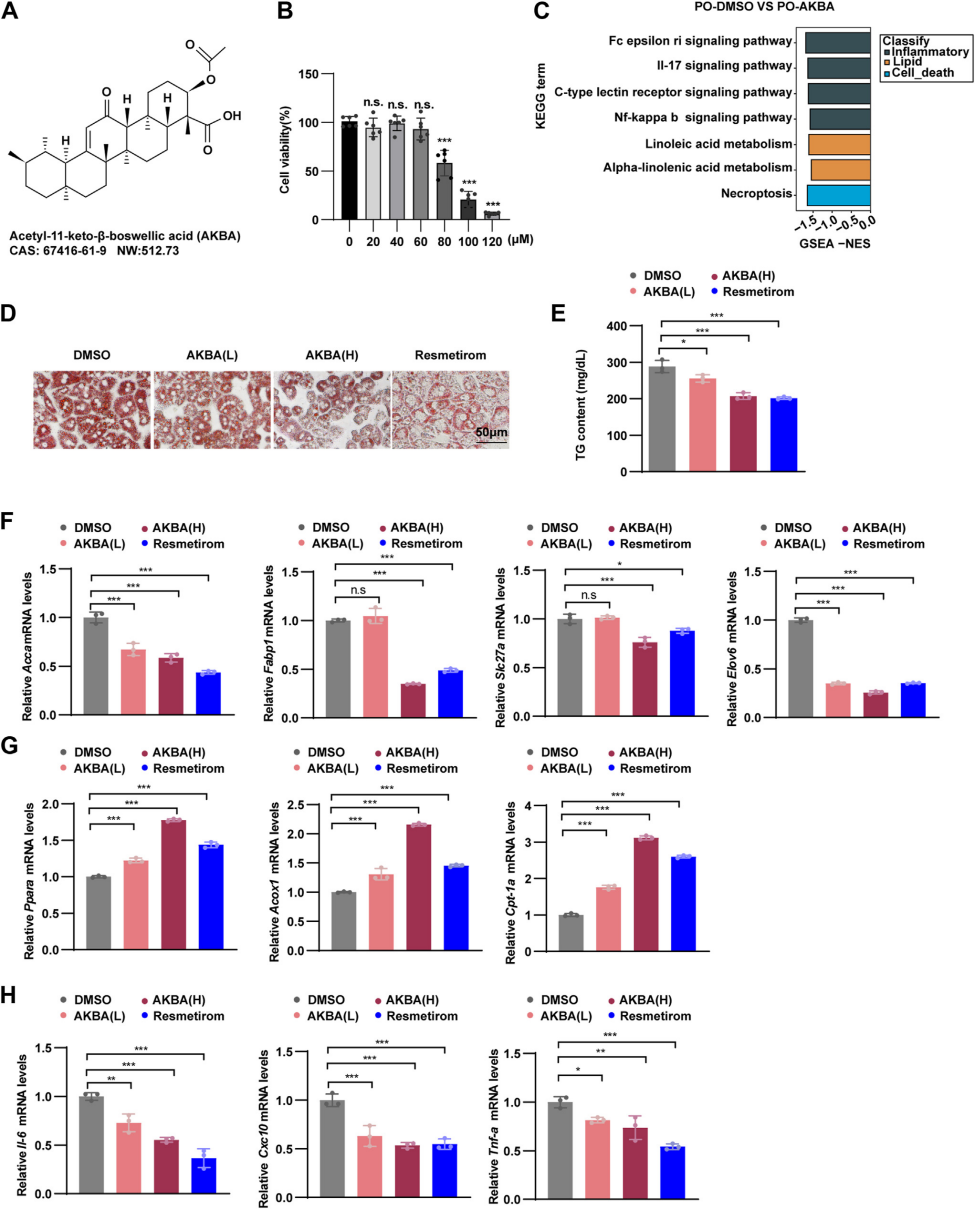
AKBA treatment reduced PO-induced lipid accumulation and inflammation in primary murine hepatocytes. A: The chemical structure of AKBA. B: Cytotoxicity of different concentrations of AKBA in primary hepatocytes. C: Pathways related to lipid metabolism, inflammation, and cell death based on GSEA. n = 3 samples per group. D: Primary hepatocytes were stained with ORO after stimulation with PO medium for 12 h with or without AKBA treatment (30 μM AKBA for low dose group, 60 μM AKBA for high dose group). Resmetirom (60 μM) was used as the positive control. Scale bar, 50 μm. E: Cellular TG levels of primary mouse hepatocytes in the indicated groups. n = 3 samples per group (30 μM AKBA for low dose group, 60 μM AKBA for high dose group). Resmetirom (60 μM) was used as positive control. F–H: mRNA levels of genes related to lipid metabolism (*Fabp-1, Slc27a, Elov6, Pparα, Acox1,* and *Cpt-1a*) and inflammation (*Il-6, Cxcl10,* and *Tnf-a*) in the indicated groups. Resmetirom (60 μM) was used as positive control. *n* = 3 samples per group. All the data are presented as the means ± SD and were analyzed by one-way ANOVA. ∗ indicates *P* < 0.05, ∗∗ indicates *P* < 0.01, ∗∗∗ indicates *P* < 0.001.

The chemical structure of AKBA is depicted in Fig. 1A. To assess its cytotoxicity, a CCK-8 assay was performed on primary hepatocytes, which revealed a concentration-dependent increase in cell toxicity beginning at 60 μM (Fig. 1B). To comprehensively investigate the molecular effects of AKBA, we conducted RNA sequencing on primary hepatocytes treated with PO, with or without 60 μM AKBA. GSEA analysis indicated that AKBA treatment downregulated key pathways associated with inflammation, lipid metabolism, and cell death (Fig. 1C).

To further evaluate the effect of AKBA on cellular lipid metabolism and inflammation, subsequent experiments utilized 30 μM and 60 μM AKBA. ORO staining analysis showed that AKBA treatment significantly reduced lipid droplet accumulation in primary hepatocytes induced by PO (Fig. 1D). This lipid-lowering effect was further confirmed by measurements of TG levels, which revealed a significant decrease in TG content following AKBA treatment (Fig. 1E). Notably, 60 μM AKBA exhibited a more pronounced effect than 30 μM AKBA, confirming the dose-dependence of AKBA's activity. Furthermore, 60 μM AKBA displayed a comparable effect to the positive control, Resmetirom. (Fig. 1D, E). Consistent with these observations, gene expression analysis confirmed that AKBA significantly modulated the expression of key metabolic and inflammatory markers. Specifically, AKBA treatment increased the expression of *Pparα*, *Acox1*, and *Cpt-1α*, key regulators of fatty acid oxidation, while concurrently reducing the expression of pro-inflammatory cytokines (*Il-6*, *Cxcl10*, and *Tnf-α*), lipid synthesis gene *Accα*, and lipid transport genes (*Fabp1, Slc27a, and Elov6*) (Fig. 1F–H). These molecular effects mirrored those observed with the positive control, Resmetirom (Fig. 1F–H), further supporting the therapeutic potential of AKBA in mitigating PO-induced metabolic disturbances in primary hepatocytes.

### AKBA alleviates insulin resistance and reduces hepatic steatosis in HFD-fed mice

**Fig. 2:**
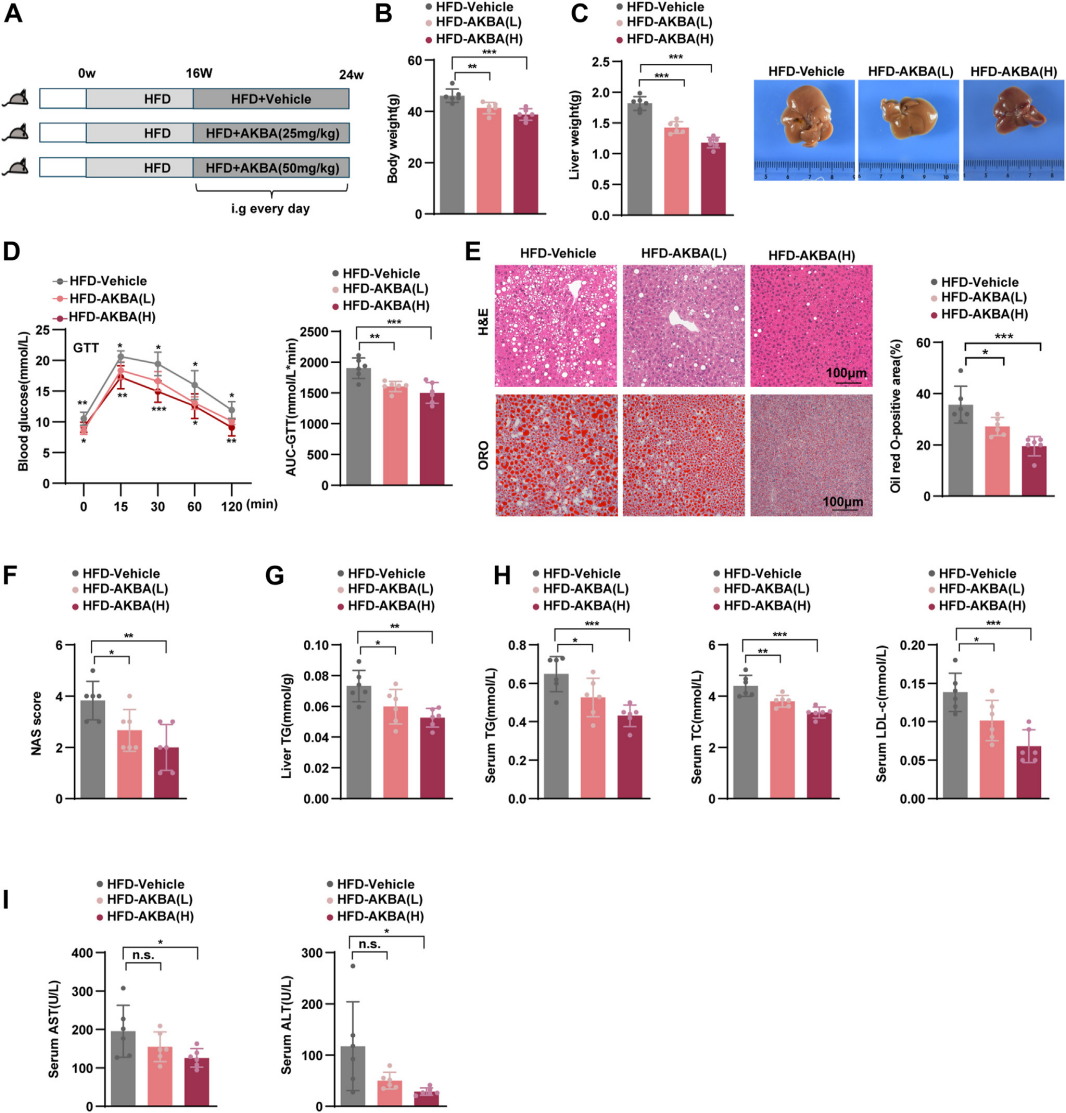
AKBA alleviated insulin resistance and hepatic steatosis in HFD-fed mice. A: The procedure was used to test the protective effects of AKBA (25 mg/kg for the low dose group; 50 mg/kg for the high dose group) on NAFLD mice. B, C: Body weight, liver weight, and representative morphology of the liver in the various groups. n = 6 per group. D: Representative GTT results of mice in the indicated groups. n = 6 per group. E: The left panel displays representative liver tissue images stained with H&E and ORO, and the right panel displays the quantification of ORO-positive areas from the indicated groups. n = 6 per group. Scale bar, 100 μm. F: NAS score in the indicated groups. n = 6 per group. G, H: liver TG content and Serum lipid (TC, TG, and LDL-c) levels were assessed in the specified mouse groups. n = 6 per group. I: Serum ALT and AST levels in the indicated groups. n = 6 per group. All the data are presented as the means ± SD and were analyzed by one-way ANOVA or Student's *t* test. n.s. indicates not significant, ∗ indicates *P* < 0.05, ∗∗ indicates *P* < 0.01, ∗∗∗ indicates *P* < 0.001.

To assess the therapeutic potential of AKBA in vivo, mice were fed an HFD for 16 weeks and then divided into three groups: a low-dose AKBA treatment group (25 mg/kg), a high-dose AKBA treatment group (50 mg/kg), and a vehicle control group. All groups remained on the HFD for an additional 8 weeks (Fig. 2A). As shown in Figure 2B, C, AKBA treatment resulted in significant reductions in both body weight and liver weight compared to the vehicle group. Furthermore, AKBA treatment notably improved glucose tolerance, as evidenced by an enhanced glucose tolerance test (Fig. 2D). Histological analysis revealed that the livers of HFD-fed mice exhibited pronounced hepatic steatosis and a high NAS score, both of which were significantly reduced following AKBA intervention (Fig. 2E, F). Subsequent biochemical analyses demonstrated that AKBA treatment markedly decreased hepatic TG content, as well as serum lipid levels, including TC, TG, and LDL-c, compared to the vehicle group (Fig. 2G, H). Moreover, serum levels of liver enzymes ALT and AST were significantly lower in the AKBA-treated group, indicating improved liver function (Fig. 2I). Notably, the efficacy of AKBA was dose-dependent, with the high-dose group exhibiting greater improvements than the low-dose group (Fig. 2B–I).

### AKBA treatment improves HFHC diet-induced NASH in mice

**Fig. 3:**
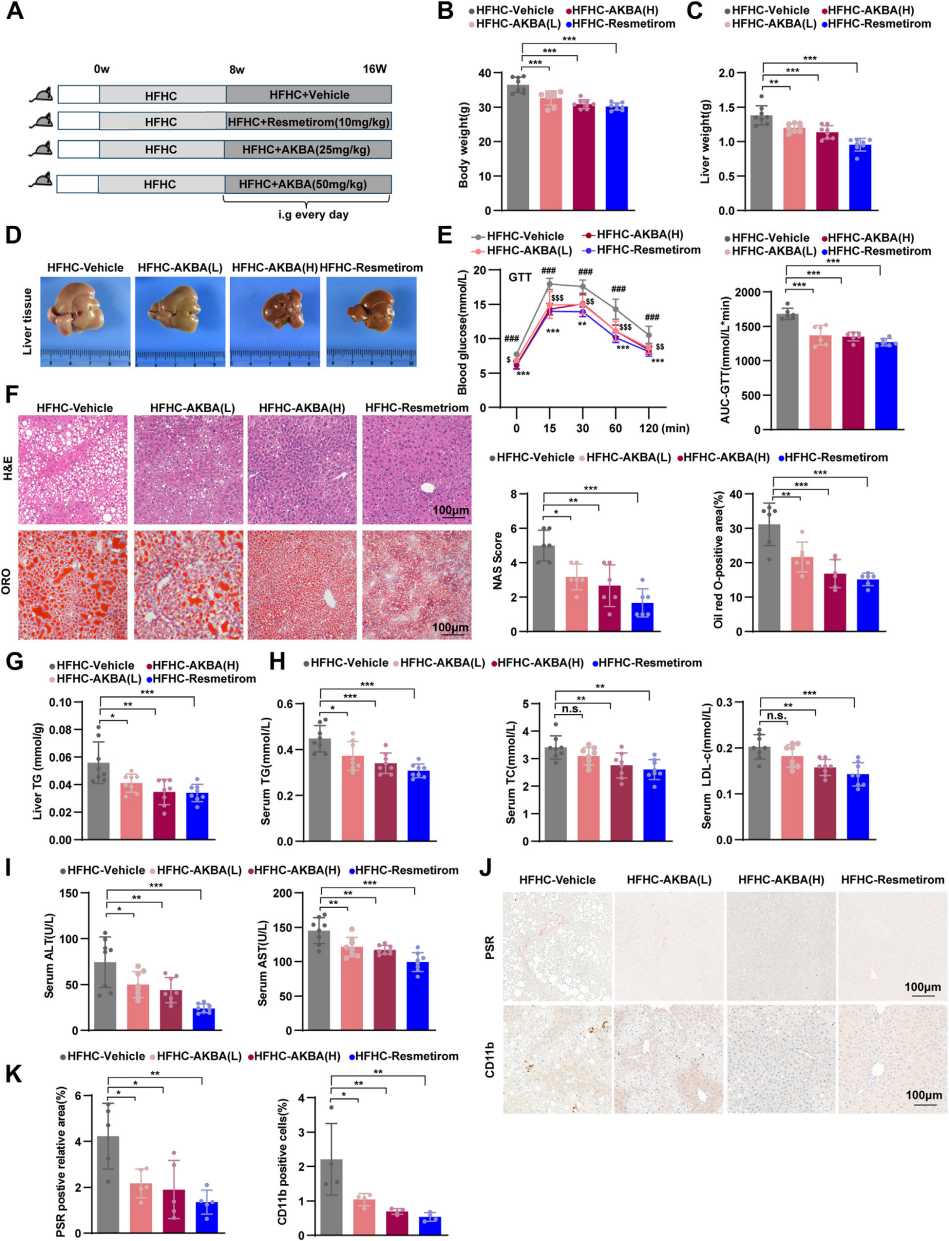
AKBA mitigated hepatic steatosis, inflammation, and fibrosis in NASH mice. A: The procedure used to test the protective effects of AKBA (25 mg/kg for high-dose group; 50 mg/kg for high-dose group) on NASH mice; Resmetirom (10 mg/kg) was the positive control group; n = 8 per group. B–D: Body weight, liver weight, and representative morphology of the liver in the indicated groups. n = 8 per group. E: Representative GTT results of mice in the indicated groups. # indicates the *P*-value between the HFHC-vehicle group and the HFHC-Resmetirom group; ∗ indicates the *P*-value between the HFHC-vehicle group and the HFHC-AKBA (H) group. ^$^ indicates the *P*-value between the HFHC-vehicle group and the HFHC-AKBA (L) group. n = 6 per group. F: The left panel displays representative H&E and ORO staining images of the indicated groups, and the right panel displays the NAS scores and the quantification of ORO-positive areas. n = 6 per group. Scale bar, 100 μm. G: TG content in the livers of the four groups. n = 8 per group. H: Serum lipid (TC, TG, and LDL-c) levels were assessed in the indicated groups. n = 8 per group. I: Serum ALT and AST activities in the indicated groups. n = 8 per group. J: Representative images of PSR and CD11b immunohistochemical staining in the indicated groups. *n* = 4–5 per group. Scale bar, 100 μm. K: Results of statistical analysis of PSR and CD11b positive areas in liver sections. *n* = 4–5 per group. All the data are presented as the means ± SD and were analyzed by one-way ANOVA or Student's *t* test. n.s. indicates not significant, ∗ indicates *P* < 0.05, ∗∗ indicates *P* < 0.01, ∗∗∗ indicates *P* < 0.001; ^###^ indicates *P* < 0.001, and ^$^ indicates *P* < 0.05, ^$$^ indicates *P* < 0.01, ^$$$^ indicates *P* < 0.001.

**Figure S1:**
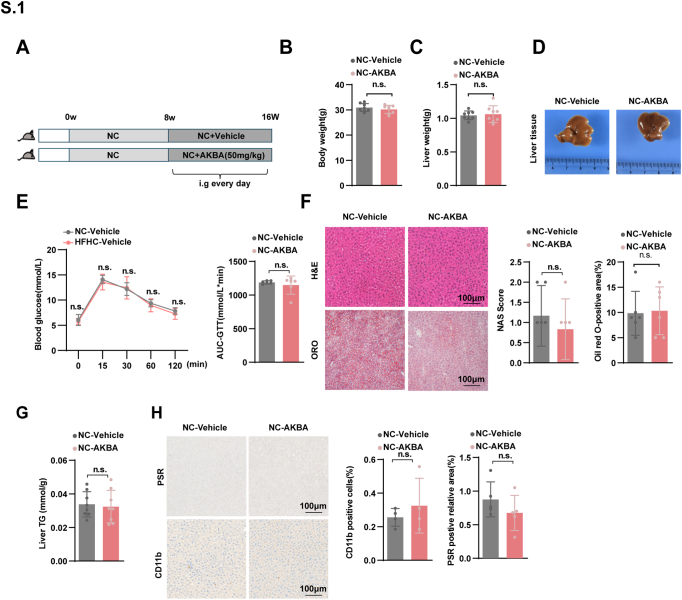


To evaluate the effects of AKBA on NASH, mice were fed a HFHC or NC diet for 8 weeks, followed by daily gavage administration of Resmetirom (10 mg/kg) or AKBA (25 mg/kg; 50 mg/kg). These treatments were administered concurrently with the HFHC or NC diet for an additional 8 weeks (Fig. 3A). AKBA treatment had no discernible effect on NASH characteristics in NC-fed mice (supplemental Fig. S1); however, it attenuated both body weight and liver weight in HFHC-fed mice compared to the vehicle group (Fig. 3B, C). Morphological examination of the liver revealed a decrease in liver volume in the AKBA-treated group, suggesting reduced hepatic congestion and steatosis (Fig. 3D). Furthermore, glucose tolerance was significantly improved in the AKBA-treated group, as demonstrated by lower glucose concentrations in the GTT test (Fig. 3E).

Histologic evaluation using the NAS system revealed a significant reduction in NAS score and lipid droplet area in the AKBA-treated group compared to the vehicle group. Notably, the effect of AKAB was comparable to that of Resmetirom, and this reduction was dose-dependent, with the higher dose exhibiting a superior effect to that of the lower dose group, as evidenced by H&E and ORO staining (Fig. 3F). Subsequent biochemical analyses showed that AKBA treatment significantly reduced hepatic TG levels and serum lipid levels, including TC, TG, and LDL-c, compared to the vehicle group (Fig. 3G, H). In addition, AKBA significantly lowered serum ALT and AST levels, indicating improved liver function (Fig. 3I).

To assess the impact of AKBA on liver fibrosis and inflammation, we examined macrophage infiltration and collagen deposition. Immunohistochemical analysis revealed a significant reduction in both macrophage infiltration and collagen deposition in the liver of AKBA-treated mice (Fig. 3J, K), suggesting a potential anti-fibrotic effect of AKBA. Together, these findings indicate that AKBA treatment alleviates HFHC diet-induced NASH by reducing hepatic steatosis, improving glucose metabolism, lowering serum lipids, and attenuating liver fibrosis and inflammation.

### AKBA inhibits NASH-associated molecular features

**Fig. 4:**
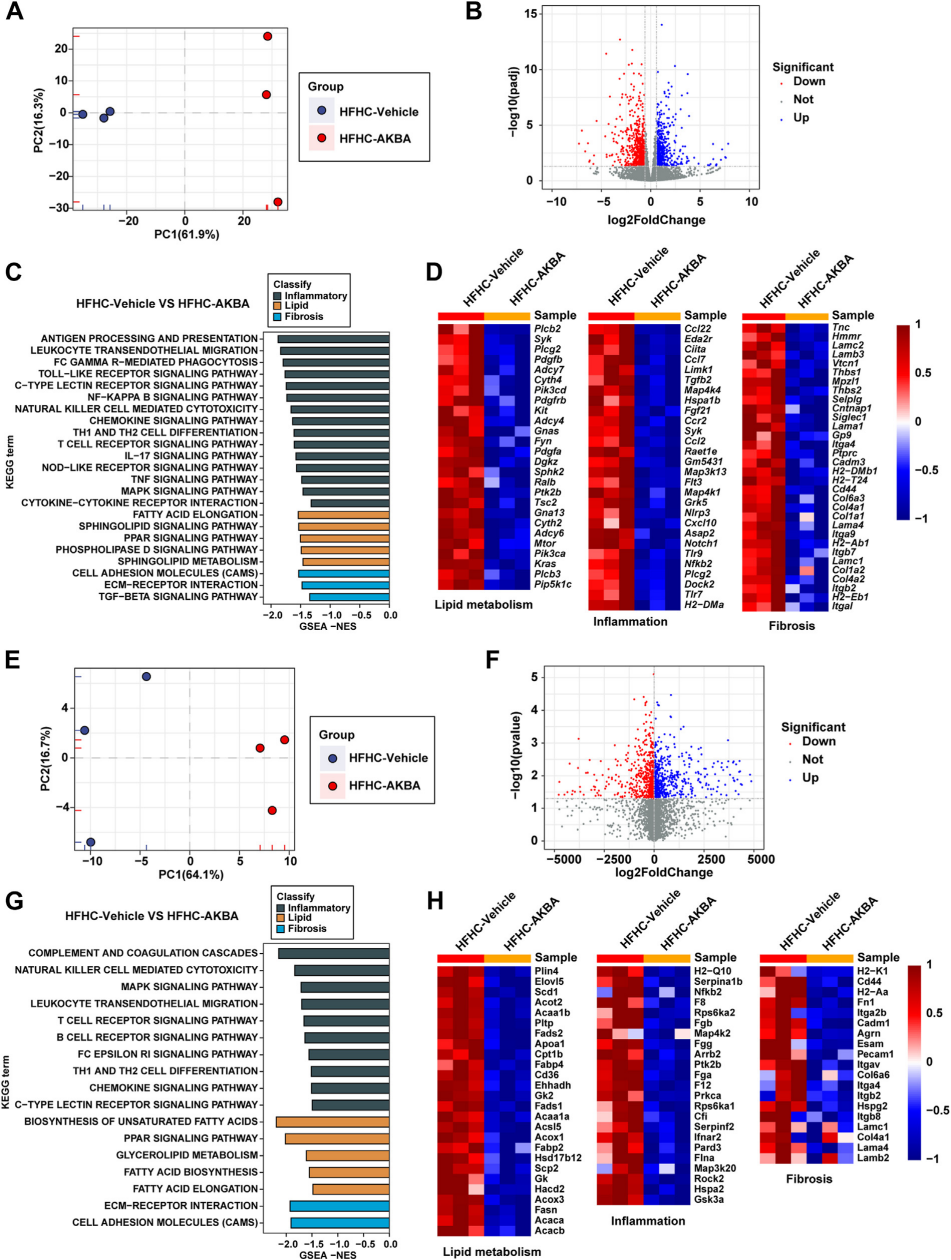
AKBA downregulated lipid metabolism, inflammation, and fibrosis pathways. A: PCA was performed on RNA-seq data obtained from the HFHC-vehicle group and the HFHC-AKBA group. *n* = 3 per group. B: Volcano map of DEGs between the HFHC-AKBA group and HFHC-vehicle group. C: GSEA of pathways related to lipid metabolism, inflammation, and fibrosis. *n* = 3 per group. D: Heatmaps displaying gene expression patterns associated with lipid metabolism, inflammation and fibrosis as determined by RNA-seq analysis. *n* = 3 per group. E: PCA was conducted on proteomic data from the HFHC-vehicle group and the HFHC-AKBA group. F: Volcano map of differentially expressed proteins between the HFHC-AKBA group and the HFHC-vehicle group. G: GSEA of pathways related to lipid metabolism, inflammation and fibrosis. *n* = 3 per group. H: Heatmaps displaying protein expression patterns associated with lipid metabolism, inflammation and fibrosis as determined by proteomic analysis. *n* = 3 per group.

To explore the molecular mechanisms underlying the protective effects of AKBA on NASH, RNA sequencing (RNA-seq) was conducted on liver samples from HFHC-fed mice in both the AKBA and vehicle groups. Unsupervised principal component analysis (PCA) revealed distinct separation between the samples from the vehicle group and the AKBA group, indicating clear molecular differences between the two conditions (Fig. 4A). Differentially expressed genes (DEGs) between the AKBA and HFHC groups were visualized using a volcano plot, highlighting significant changes in gene expression (Fig. 4B). Gene set enrichment analysis (GSEA) revealed that AKBA treatment downregulated key pathways associated with inflammation, lipid metabolism, and fibrosis (Fig. 4C). Furthermore, a heatmap generated from GSEA showed a marked downregulation of hepatic genes involved in fatty acid synthesis (e.g., *Sphk2*, *Pik3cd*) and elongation (e.g., *Plcb3*, *Cyth2*) as well as pro-inflammation genes (e,g., *Ccl22*, *CcR2*), cell adhesion molecules (e.g., *Itga4*, *Itgb7*), collagens (e.g., *Col1a2*, *Col4a2*), and other genes promoting NASH in the AKBA-treated group (Fig. 4D).

To confirm these findings at the protein level, we performed proteomic analysis on liver tissues from both the AKBA and vehicle groups following HFHC treatment. PCA of the proteomic data also demonstrated clear clustering of the samples from the two groups (Fig. 4E). The differentially expressed proteins between the AKBA and vehicle groups were visualized using a volcano plot, further confirming the significant molecular alterations induced by AKBA treatment (Fig. 4F). GSEA of the proteomic data again revealed that AKBA treatment downregulated pathways related to inflammation, lipid metabolism, and fibrosis (Fig. 4G). A heatmap based on GSEA analysis further validated the downregulation of hepatic proteins involved in fatty acid biosynthesis (e.g., Scd1, Acaca) and elongation (e.g., Elovl5, Hacd2), as well as pro-inflammation molecules (e.g., Nfkb2, Map4k2), cell adhesion molecules (e.g., Itga2b, Itga4), and collagens proteins (e.g., Col6a6, Col4a1) (Fig. 4H). Collectively, this molecular profile demonstrates that AKBA treatment leads to the downregulation of a broad spectrum of genes and proteins, particularly those involved in inflammation, lipid metabolism, and fibrosis, supporting the therapeutic potential of AKBA in ameliorating the pathological features of NASH.

### AKBA directly binds to the MGLL protein and restricts its activity

**Fig. 5:**
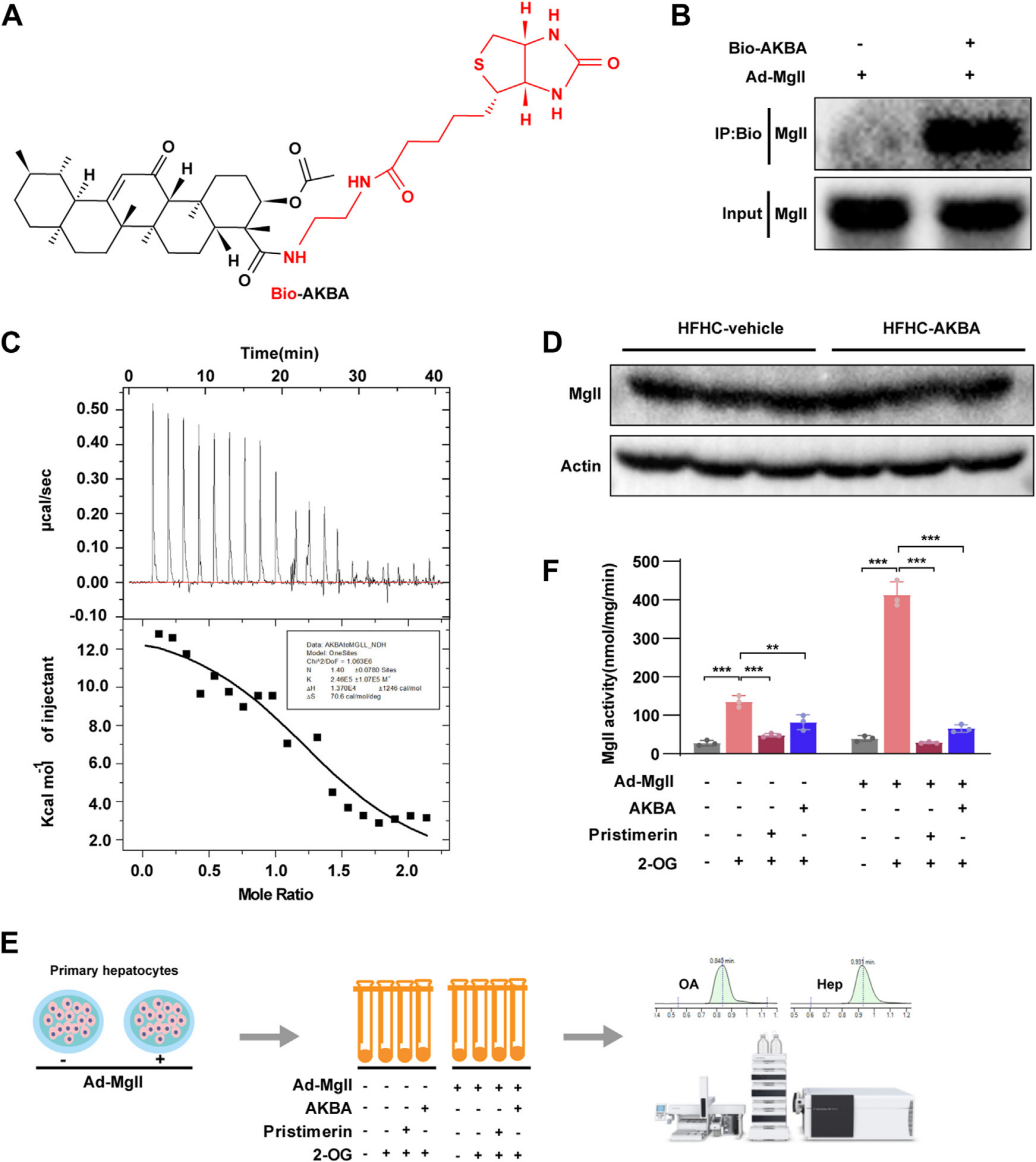
AKBA directly binds to the MGLL protein and inhibits its activity. A: Structure of Bio-AKBA. B: Streptavidin-covered beads with biotin or bio-AKBA were incubated with the cell lysates of primary hepatocytes infected with the MGLL-overexpression adenovirus for 24 h. Western blot was used to determine the levels of bound proteins and total proteins. C: The binding affinity of AKBA with MGLL was determined using ITC. D: The impact of AKBA on MGLL expression. E, F: LC‒MS-based assay for endogenous MGLL's or overexpression of MGLL's activity in the presence of AKBA (50 μM) or pristimerin (50 μM). All the data are presented as the means ± SD and were analyzed by one-way ANOVA or Student's *t* test. ∗∗ indicates *P* < 0.01, ∗∗∗ indicates *P* < 0.001.

MGLL plays a crucial role in the development of NASH by regulating lipid metabolism and inflammation in the liver. Recent studies suggest that targeting MGLL could be a promising therapeutic approach for NASH. Given that AKBA shares structural similarities with other natural compounds, pristimerin and celastrol, which are known to inhibit MGLL activity (24), we hypothesized that AKBA may also modulate MGLL activity. To test this hypothesis, we biotinylated AKBA and conducted a pull-down assay to examine its interaction with MGLL (Fig. 5A). The biotinylated AKBA (Bio-AKBA) was incubated with lysates from primary hepatocytes overexpressing MGLL, and the results demonstrated that MGLL was effectively pulled down by Bio-AKBA, indicating an interaction between AKBA and MGLL (Fig. 5B). To further investigate whether this interaction is direct, we performed isothermal titration calorimetry (ITC) to assess the binding affinity between AKBA and purified MGLL protein. The ITC analysis revealed that AKBA bound to MGLL with a dissociation constant (Kd) of 4.06 × 10^-5^ mol/L, confirming a high-affinity interaction between the two molecules (Fig. 5C).

Next, we investigated whether AKBA affects MGLL content in the liver. Western blot analysis revealed that AKBA treatment did not alter the protein levels of MGLL (Fig. 5D), suggesting that AKBA does not affect the MGLL content but may regulate its activity. To explore this further, we performed a liquid chromatography-mass spectrometry (LC-MS)-based assay to evaluate MGLL activity in the presence or absence of AKBA, using pristimerin as a positive control (Fig. 5E). The results showed that both AKBA and pristimerin significantly reduced MGLL activity in wild-type primary hepatocytes as well as in those overexpressing MGLL (Fig. 5F). These findings demonstrate that AKBA directly binds to MGLL and reduces its enzymatic activity, providing further evidence that AKBA may exert its therapeutic effects in metabolic disorders through the modulation of MGLL activity.

### Identification of the amino acid residues involved in the interaction between AKBA and MGLL

**Fig. 6:**
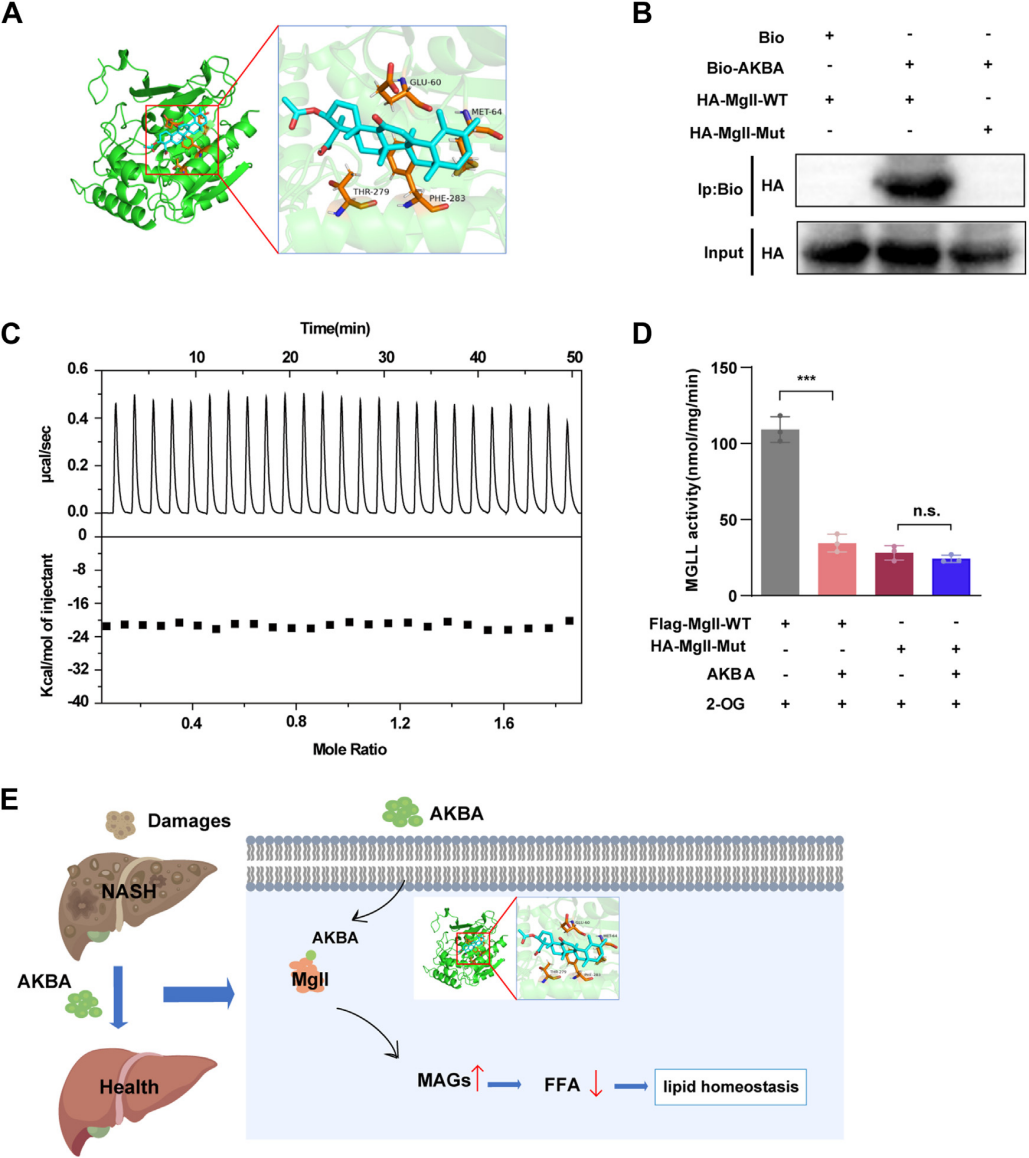
The key amino acid residues involved in the interaction between AKBA and MGLL. A: Molecular docking results of AKBA with the protein MGLL. B: Pull-down assay of Bio-AKBA with the wild-type or mutated HA-MGLL in 293T cells. C: ITC analysis of the interaction between AKBA and the mutated MGLL protein. D: The activity of wild-type or mutated MGLL in the presence of AKBA. E: Graphical abstract showing the hepatoprotective effects of AKBA. All the data are presented as the means ± SD and were analyzed by one-way ANOVA or Student's *t* test. n.s indicates not significant, ∗∗∗ indicates *P* < 0.001. AA, arachidonic acid; MAGs, monoglycerides.

To identify the critical amino acid residues involved in the interaction between AKBA and MGLL, molecular docking simulations were performed. The docking results revealed that the residues GLU60, MET64, THR279, and PHE283 may be pivotal in the binding of AKBA to MGLL (Fig. 6A). To further investigate the significance of these residues, we constructed a site-directed mutant of MGLL, with all four amino acid residues (GLU60, MET64, THR279, and PHE283) mutated, to examine whether the interaction with AKBA could be disrupted. The pull-down assay demonstrated that Bio-AKBA effectively pulled down wild-type MGLL, but not the MGLL mutant, indicating that these mutations disrupted the AKBA-MGLL interaction (Fig. 6B). ITC analysis also confirmed the loss of affinity between AKBA and the purified MGLL mutant protein, providing further evidence that these residues are crucial for the binding interaction (Fig. 6C). In addition, we assessed the enzymatic activity of the MGLL mutant and observed that it exhibited reduced activity compared to the wild-type MGLL. The presence of the MGLL mutant completely abolished the inhibitory effect of AKBA on MGLL activity, further supporting the importance of these residues in mediating AKBA's inhibitory effect on MGLL (Fig. 6D). Together, these findings demonstrate that GLU60, MET64, THR279, and PHE283 are critical for the interaction between AKBA and MGLL. A graphical abstract summarizing these findings is presented in Figure 6E.

### MGLL inhibition mediates AKBA's hepatoprotective effects

**Fig. 7:**
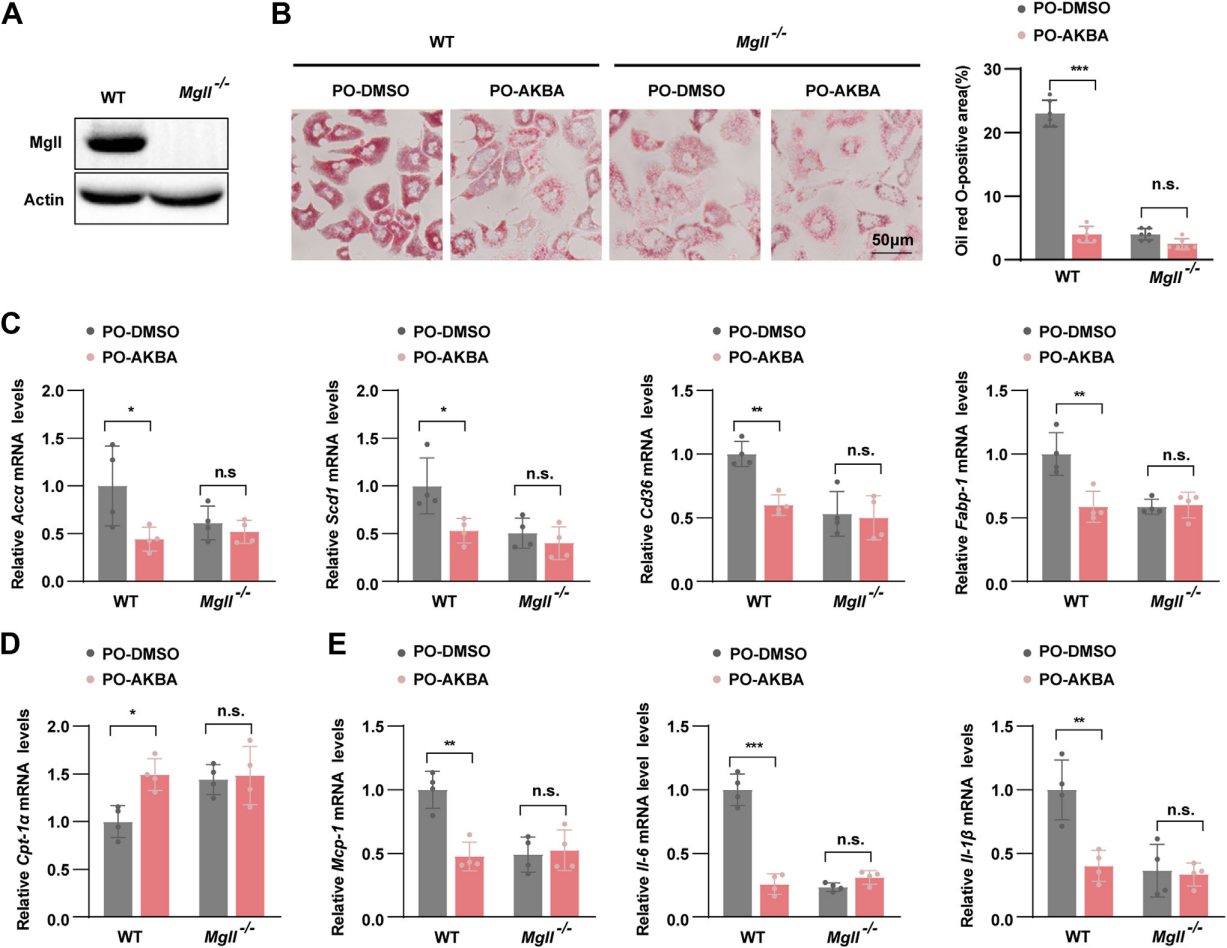
MGLL inhibition was responsible for the hepatoprotective effect of AKBA. A: Hepatic MGLL protein levels in WT and KO mice. B: representative images of ORO staining showing the degree of lipid accumulation in WT or KO primary hepatocytes stimulated with PO for 12 h in the presence of AKBA. Scale bar, 50 μm. C, D: mRNA levels of genes involved in lipid metabolism (*Accα, Scd1, Cd36, Fabp-1,* and *Cpt-1α*) and (E) and inflammatory factors (*Mcp-1, Il-1β,* and *Il-6*) in primary hepatocytes exposed to PO in the presence of AKBA. n = 4 per group. All the data are presented as the means ± SD and were analyzed by one-way ANOVA or Student's *t* test. n.s. indicates not significant, ∗ indicates *P* < 0.05, ∗∗ indicates *P* < 0.01, ∗∗∗ indicates *P* < 0.001.

To determine whether the inhibition of MGLL is essential for the hepatoprotective effects of AKBA, we isolated primary hepatocytes from both *Mgll* knockout (KO) and wild-type (WT) mice (Fig. 7A). These hepatocytes were subsequently exposed to PO to simulate conditions of lipid accumulation and inflammation. ORO staining revealed that the AKBA-mediated inhibition of lipid accumulation induced by PO was almost entirely negated by MGLL deficiency (Fig. 7B). Next, we evaluated the expression of key lipid metabolism and inflammatory genes. In WT hepatocytes, AKBA treatment resulted in a significant reduction in the expression of genes involved in fatty acid synthesis (*Accα* and *Scd1*), fatty acid uptake (*Cd36* and *Fabp-1*), and pro-inflammatory cytokines (*Mcp-1, Il-6,* and *Il-1β*) (Fig. 7C, E). Conversely, AKBA treatment led to an increase in the expression of genes associated with fatty acid β-oxidataion, notably *Cpt-1α* (Fig. 7D). However, in KO hepatocytes, the AKBA-mediated reduction in gene expression was almost completely abolished, suggesting that MGLL is critical for mediating AKBA's protective effects against lipid metabolism dysregulation and inflammation.

### Inhibition of MGLL abrogates the anti-NASH effect of AKBA

**Fig. 8:**
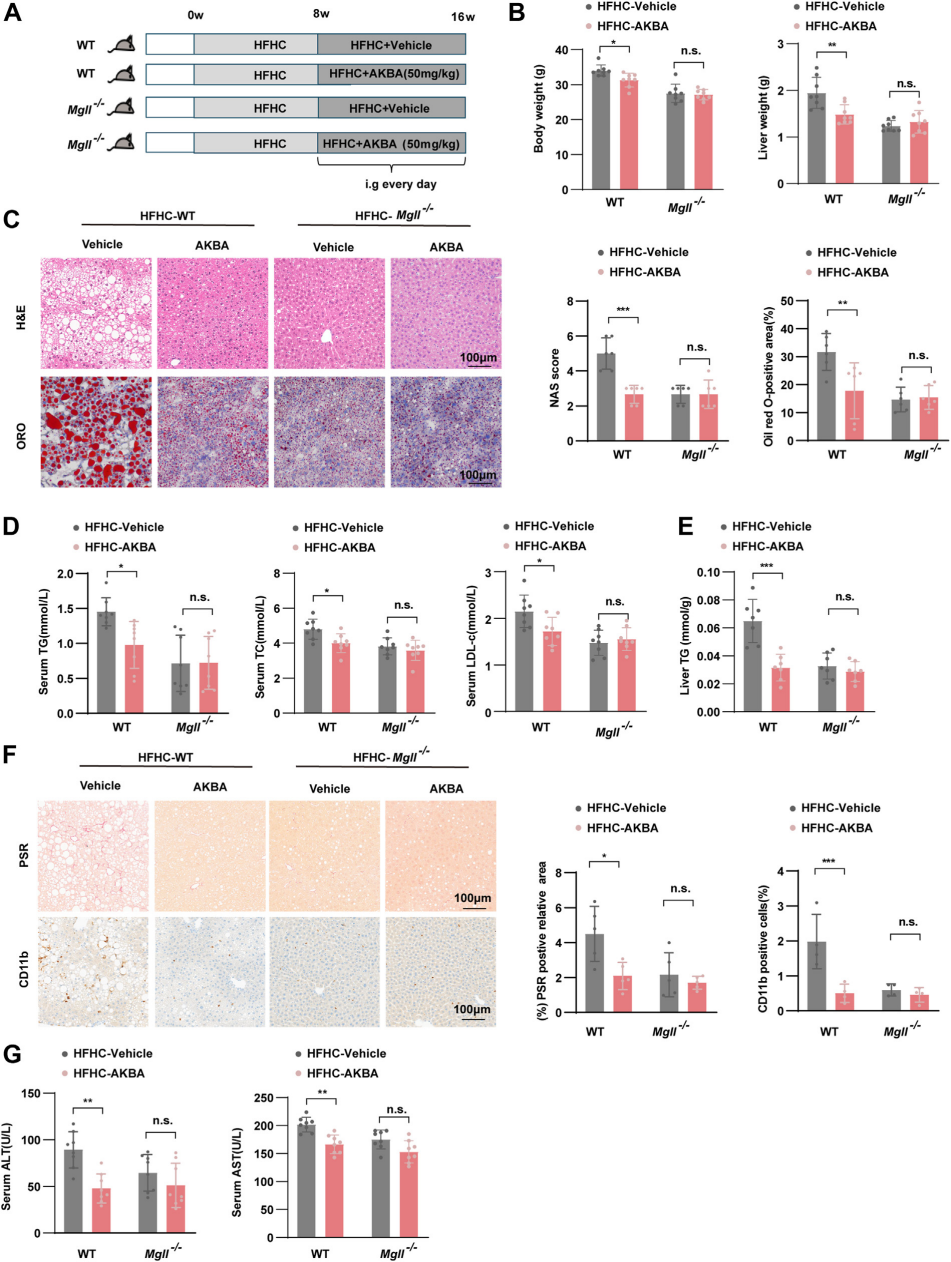
MGLL inhibition mediated the anti-NASH effect of AKBA. A: Schematic of the experimental process for assessing the impact of AKBA on WT and KO mice with NASH. n = 8 per group. B: Liver weight and body weight of the four groups. *n* = 8 per group. C: The left panel displays representative images of liver tissues stained with H&E and ORO, and the right panel displays NAFLD activity scores. The statistical results of ORO-positive areas are shown for the indicated groups. *n* = 6 per group; scale bar, 100 μm. D: Serum lipid (TC, TG, LDL-c) levels were assessed in the specified groups. *n* = 8 per group. E: Liver TG content in the various groups of mice. F: Representative images of PSR and CD11b immunohistochemical staining in the indicated groups. PSR and CD11b-positive areas in liver sections from the indicated groups; n = 4–5 per group. Scale bar, 100 μm. G: Serum ALT and AST levels in the indicated groups. *n* = 8 per group. All the data are presented as the means ± SD and were analyzed by one-way ANOVA or Student's *t* test. n.s. indicates not significant, ∗ indicates *P* < 0.05, ∗∗ indicates *P* < 0.01, ∗∗∗ indicates *P* < 0.001.

To further investigate whether the inhibition of MGLL is essential for AKBA's anti-NASH effects, we conducted in vivo experiments using both KO and WT mice. Mice were fed a HFHC diet for 8 weeks, followed by daily AKBA treatment (50 mg/kg) while continuing the HFHC diet for an additional 8 weeks (Fig. 8A). AKBA treatment significantly reduced body weight and liver weight induced by the HFHC diet in WT mice, but these beneficial effects were completely abolished in KO mice (Fig. 8B). Histological analysis showed that AKBA treatment reduced hepatic lipid accumulation, but this lipid-lowering effect was almost completely abolished in KO mice (Fig. 8C). Furthermore, AKBA treatment significantly decreased hepatic TG levels and improved serum lipid profiles, including TC, TG, and LDL-c. However, these effects were absent in KO mice (Fig. 8D, E). These results underscored the critical role of MGLL in mediating AKBA's regulatory effects on lipid metabolic dysfunction.

To assess the impact of AKBA on liver fibrosis and inflammation, we performed PSR staining to evaluate collagen deposition and CD11b immunohistochemical staining to assess inflammatory cell infiltration. AKBA treatment alleviated both liver fibrosis and hepatic inflammation in WT mice fed a HFHC diet, but these beneficial effects were abolished in KO mice (Fig. 8F). Furthermore, AKBA treatment significantly reduced serum ALT and AST levels, indicating improved liver function. This protective effect was almost completely abolished in MGLL KO mice (Fig. 8G). Collectively, the absence of MGLL abolished the protective effects of AKBA, highlighting the essential role of MGLL inhibition in AKBA's therapeutic action against NASH.

## Discussion

MGLL, a serine hydrolase primarily expressed in the liver, brain, adipose tissue, and other organs, is implicated in various metabolic disorders, inflammation, and fibrosis (25, 26). Extensive research has indicated that MGLL plays a critical role in maintaining metabolic homeostasis, with its dysregulation linked to the pathogenesis of several liver diseases (27, 28). As a result, MGLL has emerged as a promising therapeutic target for liver diseases, with recent studies highlighting its potential in NAFLD and NASH. Specifically, studies using *Mgll*^*−/−*^ mice on an HFD diet showed significant improvements in body weights, serum lipid profiles, insulin sensitivity, and hepatic steatosis compared to WT mice (29, 30, 31, 32, 33). These findings underscore the pivotal role of MGLL in regulating lipid metabolism and inflammatory processes, both of which are central to the pathophysiology of liver diseases. At the molecular level, MGLL influences two interconnected signaling pathways: the modulation of endocannabinoid levels and the regulation of triacylglycerol catabolism (34). By hydrolyzing monoacylglycerols, MGLL regulates the availability of FFAs, which in turn influences lipid storage and inflammatory responses in the liver. The inhibition of MGLL has been shown to reduce FFA production, thereby diminishing hepatic lipid accumulation and promoting anti-inflammatory effects (35). These findings highlight MGLL's critical role in maintaining metabolic balance, suggesting that its inhibition could represent an effective therapeutic strategy for treating metabolic disorders, particularly those associated with hepatic dysfunction.

In this study, in vitro experiments demonstrated that AKBA effectively mitigated lipid accumulation and inflammation in primary hepatocytes under PO-induced stress. In vivo AKBA treatment significantly alleviated liver injury, steatosis, inflammatory cell infiltration, and fibrosis in a NASH animal model. Notably, we identified MGLL as a key target through which AKBA exerts its beneficial effects. Our investigation revealed that mutations in residues GLU60, MET64, THR279, and PHE283 of MGLL reduced its enzymatic activity, suggesting that these regions may be important for MGLL's catalytic function. Previous studies have highlighted the catalytic ternary structure of MGLL, with key residues such as Ser122, Asp239, and His269 involved in enzyme catalysis, while Cys201, Cys208, and Cys242 stabilize the active conformation of the enzyme (36, 37). These findings suggest the potential to identify additional active sites in MGLL, although further research is needed to validate these hypotheses.

Various irreversible MGLL inhibitors, such as JZL184, O-hexafluoroisopropyl carbamates, MJN110, JJKK-048, and ABX-1431, have been developed by academic and industrial groups (38, 39, 40, 41, 42, 43). However, the long-term use of such inhibitors may lead to functional antagonism or pharmacological tolerance, undermining their therapeutic potential, particularly in anti-nociceptive efficacy. To mitigate these issues, reversible inhibitors derived from naturally occurring terpenoids, including pristimerin, euphol, and andrographolide (AGP) have been proposed as safer alternatives (24, 44). Despite their promise, the in vivo effects of these compounds remain insufficiently understood, necessitating further studies to assess their efficacy and safety. Notably, compounds like ABX-1431 and AGP have already entered clinical trials, although not specifically for NASH treatment.

AKBA, a bioactive compound isolated from *Boswellia serrata*, has long been utilized in traditional Chinese medicine for its anti-inflammatory, anti-infective, anti-tumor, antioxidant, and anti-aging properties (45, 46, 47, 48). Moreover, AKBA has shown neuroprotective effects and is well-tolerated in clinical settings, particularly in treating osteoarthritis (49, 50). Given its favorable safety profile, AKBA presents a promising therapeutic candidate for NASH, a disease for which effective treatments remain limited. This study provides compelling evidence that AKBA, through its inhibitory effects on MGLL, mitigates hepatic metabolic dysfunction associated with NASH. By reducing hepatic lipid accumulation, inflammation, and fibrogenesis, AKBA demonstrates potential as a novel therapeutic approach for NASH management. In addition to its effects on MGLL activity, AKBA's hepatoprotective effects may also be linked to the anti-inflammatory action of fibroblast growth factor 21 (FGF21), which inhibits the ALOX15/15-HETE pathway and mitigates the pro-inflammatory response (51). Furthermore, similar to tetramethylpyrazine (TMP), which enhances antioxidant defenses through increased superoxide dismutase (SOD) activity and reduces oxidative stress by lowering malondialdehyde (MDA) levels (52), AKBA may also contribute to modulating oxidative stress, a key factor in the pathogenesis of liver injury. Given the critical involvement of hepatic inflammation and oxidative stress in the pathogenesis of various liver injuries, we believe AKBA holds promise as a therapeutic agent for conditions beyond NAFLD/NASH, such as liver transplantation and ischemia-reperfusion injury. Moreover, the identification of MGLL as a key target for AKBA further strengthens the rationale for developing MGLL inhibitors as a therapeutic strategy for liver diseases. With its well-established safety profile and promising therapeutic effects, AKBA warrants further clinical investigation as a potential treatment for NASH, offering hope for better management of this increasingly prevalent liver disorder.

We acknowledge that a limitation of our study is its exclusive focus on liver tissue, with the potential contributions of other tissues to the observed improvement in fatty liver remaining unexplored. While weight loss may contribute to the reduction in liver fat, our in vitro experiments provide strong evidence that AKBA exerts a direct effect on hepatocytes, suggesting that the improvement in fatty liver is primarily mediated through liver-specific mechanisms. Thus, although weight loss may play a role, our data highlight the critical importance of AKBA's direct action on liver cells in regulating lipid metabolism. These findings offer a foundation for future studies to investigate the precise mechanisms by which AKBA modulates liver fat and explore whether systemic effects also contribute to its actions.

In conclusion, this study highlights the therapeutic potential of AKBA in ameliorating NASH through the inhibition of MGLL activity. The findings not only provide insights into the molecular mechanisms underlying AKBA's effects but also underscore the importance of targeting MGLL in the treatment of hepatic metabolic disorders. As such, AKBA holds promise for advancing treatment strategies for NASH and potentially other metabolic diseases.

## Data availability

Data will be made available on request.

## Supplemental data

This article contains supplemental data.

## Conflict of interest

The authors declare that they have no conflicts of interest with the contents of this article.

## Figures and Tables

**Figure S2:**